# Impaired Meningeal Lymphatics and Glymphatic Pathway in Patients with White Matter Hyperintensity

**DOI:** 10.1002/advs.202402059

**Published:** 2024-05-05

**Authors:** Ying Zhou, Rui Xue, Yifei Li, Wang Ran, Yuping Chen, Zhongyu Luo, Kemeng Zhang, Ruoxia Zhang, Junjun Wang, Mengmeng Fang, Cong Chen, Min Lou

**Affiliations:** ^1^ Department of Neurology the Second Affiliated Hospital Zhejiang University School of Medicine Hangzhou 310009 China; ^2^ Department of Radiology the Second Affiliated Hospital Zhejiang University School of Medicine Hangzhou 310009 China

**Keywords:** glymphatic pathway, meningeal lymphatics, neuroinflammation, white matter hyperintensity

## Abstract

White matter hyperintensity (WMH) represents a critical global medical concern linked to cognitive decline and dementia, yet its underlying mechanisms remain poorly understood. Here, humans are directly demonstrated that high WMH burden correlates with delayed drainage of meningeal lymphatic vessels (mLVs) and glymphatic pathway. Additionally, a longitudinal cohort study reveals that glymphatic dysfunction predicts WMH progression. Next, in a rat model of WMH, the presence of impaired lymphangiogenesis and glymphatic drainage is confirmed, followed by elevated microglial activation and white matter demyelination. Notably, enhancing meningeal lymphangiogenesis through adeno‐associated virus delivery of vascular endothelial growth factor‐C (VEGF‐C) mitigates microglial gliosis and white matter demyelination. Conversely, blocking the growth of mLVs with a VEGF‐C trap strategy exacerbates these changes. The findings highlight the role of mLVs and glymphatic pathway dysfunction in aggravating brain white matter injury, providing a potential novel strategy for WMH prevention and treatment.

## Graphical abstract

Patients with a high burden of white matter hyperintensities are found to have impaired drainage in the meningeal lymphatic vessels and glymphatic pathway. Enhanced meningeal lymphatic vessels and glymphatic pathway drainage via adenoviral delivery of vascular endothelial growth factor‐C ameliorate white matter damage. Improvement of meningeal lymphatic vessels and glymphatic pathway function may offer a promising strategy for delaying or even preventing the progression of white matter hyperintensities.

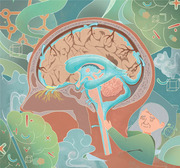

## 1 Introduction

White matter hyperintensity (WMH) is a prevalent manifestation of cerebral small vessel disease (CSVD), affecting approximately 11–21% of individuals aged 64 years and escalating to 64–94% by the age of 82.^[^ ^1^, ^2^ ^]^ As the most common brain imaging feature of CSVD, WMH poses a substantial functional impact, leading to increased cognitive decline, stroke risk, and neuropsychological disorders. Consequently, it has emerged as a pressing global medical concern.^[^ ^3^, ^4^ ^]^ However, the development of effective prevention and treatment strategies remains challenging due to its heterogeneous etiologies, diverse cerebrovascular mechanisms, and multifactorial pathologies. Therefore, an urgent demand exists for a comprehensive and novel understanding of WMH.

The intriguing discovery of meningeal lymphatic vessels (mLVs) and the glymphatic pathway has generated significant excitement, challenging the long‐standing belief that the central nervous system (CNS) lacks a conventional lymphatic system.^[^ ^5^, ^6^, ^7^ ^]^ Recent studies have unequivocally demonstrated that the cerebrospinal fluid (CSF) in the subarachnoid and cisternal spaces flows specifically through periarterial spaces, where it undergoes exchange with interstitial fluid facilitated by aquaporin‐4 water channels. Subsequently, this fluid is drained through the mLVs, or lymphatics in the cribriform plate, as well as in the perineurium of the cranial nerves, before ultimately reaching the deep cervical lymph nodes.^[^ ^5^, ^8^, ^9^ ^]^ Through the glymphatic pathway, waste products and immune cells are effectively cleared from the brain. The existence of this CSF circulation system, particularly the mLVs, presents a promising therapeutic target for various neurodegenerative and neuroinflammatory disorders.^[^ ^10^, ^11^, ^12^ ^]^

In our recent clinical observation, we discovered a correlation between the severity of WMH lesions and the function of the glymphatic pathway, using an indirect method known as the diffusion tensor image analysis along the perivascular space (DTI‐ALPS) index.^[^ ^13^ ^]^ Additionally, an animal study employing spontaneously hypertensive rats (SHR), a commonly used model for mimicking WMH pathology, demonstrated decreased glymphatic function compared to the wild‐type Wistar‐Kyoto rats (WKY).^[^ ^14^ ^]^ In general, neuroinflammation is recognized as a key pathological feature of WMH.^[^ ^15^ ^]^ Increased microglial activation in WMH lesions has been observed through ^[11C]^PK11195 positron emission tomography imaging.^[^ ^16^ ^]^ Furthermore, numerous studies have reported elevated peripheral inflammation markers in patients with WMH, including C‐reactive protein, interleukin‐6, E‐selectin, P‐selectin, vascular cell adhesion molecule‐1, and others.^[^ ^17^ ^]^ Overall, the existence of mLVs and glymphatic pathway establishes a possible connection between the peripheral immune system and central neuroinflammation by facilitating the clearance of immune cells and maintaining brain homeostasis under normal physiological conditions. Nevertheless, the direct involvement of impaired drainage function in mLVs and the glymphatic pathway in the pathogenesis and progression of WMH remains to be fully elucidated.

In this study, we investigated the direct association between the volume of WMH lesions and the drainage function of mLVs and glymphatic pathway by conducting Glymphatic MRI after intrathecal injection of a contrast agent in patients with indications for lumbar puncture. Additionally, we examined how defects in this drainage system, as reflected by the ALPS index, influenced the growth of WMH in a large longitudinal cohort. Our findings revealed a significant impairment of glymphatic function in the WMH high burden group, suggesting a connection between WMH volume and the efficiency of the glymphatic system. Furthermore, we observed that individuals with a relatively well‐functioning glymphatic system exhibited slower growth of WMH lesions over time. In a subsequent experiment, we conducted prophylactic interventions aiming at restoring mLVs drainage in aged SHR using viral delivery of vascular endothelial growth factor C (VEGF‐C). The results demonstrated that this intervention effectively alleviated microglial gliosis and white matter demyelination. Conversely, impaired mLVs drainage in aged WKY using a VEGF‐C trap strategy exacerbated inflammation and demyelination in the white matter.

## 2 Results

### 2.1 High WMH Burden Was Associated with Delayed Drainage of mLVs and Glymphatic Pathway as Visualized by Glymphatic MRI

Glymphatic MRI, a DCE‐MRI technique involving the intrathecal injection of gadolinium contrast agent, allowed visualization of the drainage of mLVs and glymphatic pathway.^[^ ^18^, ^19^ ^]^ During a study period from April 2018 to June 2022, 54 patients underwent Glymphatic MRI scan to evaluate their mLVs and glymphatic function. These patients were referred for lumbar puncture due to peripheral neuropathy, suspected CSF leakage or motor neuron disease. The study group included 27 males with a mean age of 57 years and 27 females with a mean age of 59 years. Systolic pressure averaged 145 ± 42 mmHg, diastolic pressure averaged 85 ± 12 mmHg, and intracranial pressure averaged 135 ± 89 mmH_2_O. Demographics and basic clinical data are shown in Table S1 (Supporting Information).

**Figure 1:**
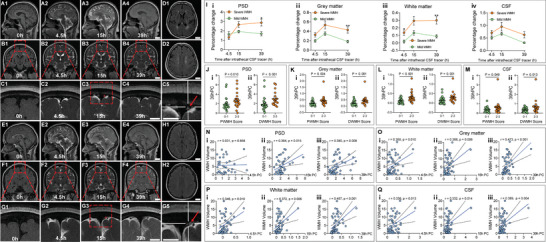
Delayed drainage of the glymphatic pathway associated with the severity of white matter hyperintensity (WMH) in patients who underwent Glymphatic MRI. A–H) Representative images showing the glymphatic pathway before and after intrathecal administration of gadodiamide in two patients with severe WMH (A–D, one individual) and mild WMH (E–H, another individual). Axial 2D‐T2‐fluid‐attenuated inversion recovery (FLAIR) images show D1,H1) their corresponding periventricular white matter hyperintensity (PWMH) and D2,H2) deep white matter hyperintensity (DWMH), respectively, with different severity. Glymphatic pathway is visualized at baseline (0 h), 4.5, 15, 39 h after intrathecal administration of gadodiamide on A,E) 3D‐T1 imaging and B,F) coronal 2D‐T2‐FLAIR imaging, with their corresponding magnified images on C and G. Of note, C5 and G5 further present the magnified images of the red dashed box on C3 and G3, respectively. Regions of interest (small red circles on C3 and G3) and parasagittal dura (PSD, red arrows on C5 and G5) are marked. I) Percentage change (PC) of signal unit ratio at different time points in PSD (i), grey matter (ii), white matter (iii) and cerebrospinal fluid (CSF) (iv) (severe WMH: *n* = 27, mild WMH: *n* = 27, two‐way ANOVA with Bonferroni's post hoc test). J–M) Comparison of the 39h PC value of J‐i) PSD, K‐i) grey matter, L‐i) white matter and M‐i) CSF between groups of PWMH score of 0–1 (*n* =  30) and 2‐3 (*n* =  24) (Mann‐Whitney U test). Comparison of the 39h PC value of J‐ii)) PSD, K‐ii) grey matter, L‐ii) white matter and M‐ii)) CSF between groups of DWMH score of 0–1 (*n* =  33) and 2–3 (*n* = 21) (Mann‐Whitney U test). N–Q) Correlation between the WMH volume and the PC value in N) PSD, O) grey matter, P) white matter, and Q) CSF from baseline to 4.5h (i), 15h (ii), and 39h (iii) (*n* = 54, Pearson correlation analysis). Scale bar = 2 cm (A1‐4, B1‐4, D1‐2, E1‐4, F1‐4, H1‐2); Scale bar = 1 cm (C1‐5, G1‐5). All error bars represent mean ± s.e.m. *p < 0.05, **p < 0.01.

**Figure** **1**A–H illustrates representative Glymphatic MRI images depicting the glymphatic pathway in two individuals with varying degrees of WMH severity. We measured the percentage change (PC) of the signal unit ratio from baseline to 4.5 hours (h), 15h, and 39h in various brain regions, including the parasagittal dura (PSD), white matter, grey matter and CSF, and the PC from baseline to 39h was quantified as the drainage function of the glymphatic pathway.^[^ ^20^, ^21^ ^]^ The PC values in the examined regions were higher in severe WMH group compared to the mild WMH group, which were categorized based on the median WMH volume of 3.77 mL (Figure 1I). Consistently, the PC values from baseline to 39h in the examined regions were higher in the periventricular WMH (PWMH) score and deep WMH (DWMH) score of 2–3 than 0–1 group (Figure 1J–M). Moreover, there was a statistically significant association between the PC values from baseline to 39h in these regions and WMH volume (*p* < 0.05 for all). When adjusting for age, gender, and hypertension, linear regression revealed that the PC values from baseline to 39h in these regions independently correlated with WMH volume (*p* < 0.05 for all). The relationship between WMH volume and PC values from baseline to 4.5, 15, and 39 h is presented in Figure 1N–Q and Table S2 (Supporting Information).

In addition, in patients who underwent Neck T1 fat‐suppression imaging, we measured the PC values of the signal unit ratio in the deep cervical lymph nodes (dCLNs), finding there was a statistically significant association between the PC values from baseline to 39 h and WMH volume (*r* = 0.629, *p* = 0.021; Figure S5, Supporting Information), indicating impaired drainage of mLVs to dCLNs with higher WMH burden.

### 2.2 Decreased Glymphatic Clearance Was Associated with WMH Volume and Progression in a Large Cohort Cross‐Sectionally and Longitudinally

**Figure 2:**
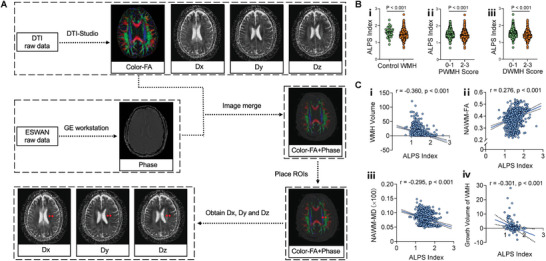
Glymphatic function associated with the presence, severity, and growth of white matter hyperintensity (WMH) in CIRCLE cohort. A) Schematic diagram illustrating the diffusion tensor image analysis along the perivascular space (DTI‐ALPS) index measurements. B) Comparison of glymphatic function, measured by the ALPS index, between WMH patients (*n* =  1149) and controls (*n* =  48) (i), periventricular white matter hyperintensity (PWMH) score of 0–1 (*n* =  523) and 2–3 (*n* =  626) groups (ii), and deep white matter hyperintensity (DWMH) score of 0–1 (*n* =  588) and 2–3 (*n* =  561) groups (iii) (Unpaired t test). C) Correlation analysis between the ALPS index and various parameters involving WMH including WMH volume (*n* =  1149) (i), normal appearing white matter‐fractional anisotropy (NAWM‐FA) (*n* =  1149) (ii), normal appearing white matter‐mean diffusivity (NAWM‐MD) (*n* =  1149) (iii), and growth volume of WMH in patients with repeat T2‐fluid‐attenuated inversion recovery (T2‐FLAIR) scanning at an average scan interval of 1.23 ± 0.51 years (*n* =  191) (iv) (Pearson correlation analysis). All error bars represent mean ± s.e.m.

Despite the potential advantage of glymphatic MRI, its widespread clinical application is limited by the requirement of lumbar puncture with intrathecal‐administered contrast agent. The ALPS index provides a promising noninvasive alternative based on diffusion tensor imaging (DTI) to evaluate the glymphatic function (**Figure** **2**A).^[^ ^13^ ^]^ Therefore, to further investigate the association between cerebral glymphatic function and WMH, we enrolled a cohort of patients with WMH and control participants who underwent DTI as a part of the CIRCLE study (ClinicalTrials.gov ID: NCT03542734). A total of 1149 WMH patients and 48 controls without WMH were included in the study, spanning from January 2010 to June 2022. Demographics and basic clinical information for the participants are provided in Table S3 (Supporting Information).

Compared to the control group, patients with WMH were found to be older (61.92 ± 8.46 vs 54.23 ± 7.01, *p* < 0.001) and had lower ALPS index (1.49 ± 0.24 vs 1.61 ± 0.26, *p* < 0.001), indicating decreased glymphatic clearance (Figure 2B–i). After adjusting for age, the ALPS index was independently associated with the presence of WMH (OR = 0.297, 95%CI [0.093‐0.954], *p* = 0.041).

Among patients with WMH, the groups with a PWMH or DWMH core of 2–3 had higher ALPS index compared to the 0–1 group (both *p* < 0.001; Figure 2B‐ii,iii). The ALPS index showed a significant negative correlation with WMH volume (*r* = ‐0.360, *p* < 0.001; Figure 2C‐i). Additionally, there was a favorable correlation between the ALPS index and the fractional anisotropy (FA) value of normal‐appearing white matter (NAWM) (*r* = 0.276, *p* < 0.001; Figure 2C‐ii). Furthermore, the ALPS index exhibited a negative correlation with the mean diffusivity (MD) value of NAWM (*r* = −0.295, *p* < 0.001; Figure 2C‐iii). Linear regression revealed that a higher ALPS index was associated with smaller WMH volume, higher NAWM‐FA and lower NAWM‐MD after adjusting for age, hypertension, hyperlipidemia, diabetes, alcohol drinking, smoking, and cerebral ventricle volume (*β* = −0.217, *p* < 0.001; *β* = 0.239, *p* < 0.001; *β* = −0.269, *p* < 0.001; Table S4, Supporting Information).

To investigate the potential involvement of the ALPS index in the progression of WMH, we calculated the growth volume of WMH in 191 WMH patients who underwent repeated T2‐fluid attenuated inversion recovery (T2‐FLAIR) scanning at follow‐up. The average scan interval was 1.23 ± 0.51 years. Detailed demographic and clinical data of the follow‐up patients are provided in Table S5 (Supporting Information). Among these patients, the growth volume of WMH showed a negative association with the baseline ALPS index (*r* = ‐0.301, *p* < 0.001; Figure 2C‐iv); Table S9, Supporting Information). After adjusting for age, cerebral ventricle volume and follow‐up interval, lower baseline ALPS index was independently associated with a greater growth volume of WMH (*β* = ‐0.203, *p* = 0.007; Table S6, Supporting Information).

### 2.3 Impaired Lymphangiogenesis and Delayed Drainage of mLVs and Glymphatic Pathway in SHR

**Figure 3:**
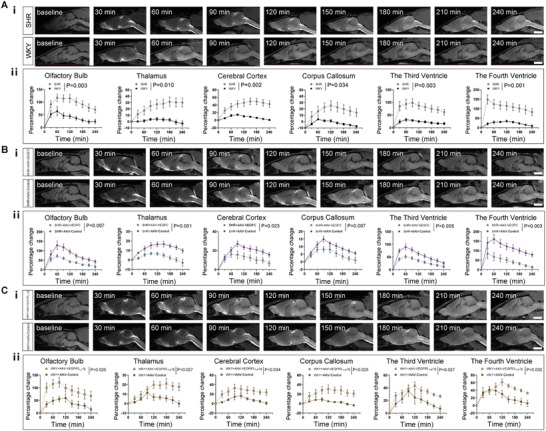
Quantitative assessments of drainage function of glymphatic pathway after intra‐cisterna magna administration of gadodiamide in rats. A‐i–C‐i) Representative 3D‐T1 images showing the glymphatic pathway at baseline and every 30 min after intra‐cisterna magna administration of gadodiamide in spontaneously hypertensive rats (SHR) versus Wistar‐Kyoto rats (WKY) (A‐i), in AAV2/9‐CMV‐rVEGF‐C treated SHR (SHR+AAV‐VEGFC) and AAV2/9‐control treated SHR (SHR+AAV‐Control) (B‐i), and in AAV2/9‐CMV‐rVEGFR3_1‐4_‐Ig treated WKY (WKY+AAV‐VEGFR3_1‐4_‐Ig) and AAV2/9‐control treated WKY (WKY+AAV‐Control) (C‐i). Scale bar = 5 mm. A‐ii–C‐ii) Comparison of glymphatic function in predefined six brain regions, including olfactory bulb, thalamus, cerebral cortex, corpus callosum, the third ventricle and the fourth ventricle between SHR (*n* = 19) and WKY (*n* = 12) (A‐ii), between SHR+AAV‐VEGFC (*n* = 9) and SHR+AAV‐Control (*n* = 8) (B‐ii), between WKY+AAV‐VEGFR3_1‐4_‐Ig (*n* = 6) and WKY+AAV‐Control (*n* = 5) (C‐ii). Data pooled from three independent experiments. All error bars represent mean ± s.e.m. *P* values were calculated by repeated‐measures two‐way ANOVA with Bonferroni's post hoc test.

**Figure 4:**
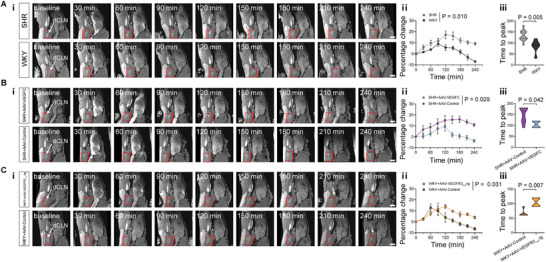
Quantitative assessments of drainage function of meningeal lymphatic vessels (mLVs) to deep cervical lymph nodes (dCLNs) after intra‐cisterna magna administration of gadodiamide in rats. Ai–Ci) Representative 3D‐T1 images showing the drainage of mLVs to dCLNs at baseline and every 30 min after intra‐cisterna magna administration of gadodiamide A‐i) in spontaneously hypertensive rats (SHR) and Wistar‐Kyoto rats (WKY), B‐i) in AAV2/9‐CMV‐rVEGF‐C treated SHR (SHR+AAV‐VEGFC) and AAV2/9‐control treated SHR (SHR+AAV‐Control), and C‐i) in AAV2/9‐CMV‐rVEGFR3_1‐4_‐Ig treated WKY (WKY+AAV‐VEGFR3_1‐4_‐Ig) and AAV2/9‐control treated WKY (WKY+AAV‐Control). The red rectangular box indicates the location of the dCLNs at each time point, and the white arrows point to the dCLNs. Scale bar = 5 mm. A‐ii–C‐ii) Comparison of percentage change of drainage from mLVs to dCLNs between SHR (*n* = 11) and WKY (*n* = 5) (A‐ii), between SHR+AAV‐VEGFC (*n* = 4) and SHR+AAV‐Control (*n* = 8) (B‐ii), between WKY+AAV‐VEGFR3_1‐4_‐Ig (*n* = 4) and WKY+AAV‐Control (*n* = 5) (C‐ii). A‐iii–C‐iii) Comparison of the average time to peak value of the dCLNs between SHR (*n* = 11) and WKY (*n* = 5) (A‐iii), between SHR+AAV‐VEGFC (*n* = 4) and SHR+AAV‐Control (*n* = 8) (B‐iii), between WKY+AAV‐VEGFR3_1‐4_‐Ig (*n* = 4) and WKY+AAV‐Control (*n* = 5) (C‐iii). Data pooled from two independent experiments. All error bars represent mean ± s.e.m. P values were calculated by repeated‐measures two‐way ANOVA with Bonferroni's post hoc test (A‐ii–C‐ii) or two‐tailed unpaired Student's t‐test (A‐iii–C‐iii).

To validate the findings observed in patients with WMH and gain mechanistic insights, we employed SHR as an animal model of WMH, with WKY serving as the control (both at 26 weeks).^[^ ^22^ ^]^ Indirect blood pressure measurements confirmed significantly higher systolic blood pressure levels in SHR compared to WKY (194 ± 27.63 mmHg vs 135 ± 23.96 mmHg, *p* < 0.001). We then assessed the drainage function of the glymphatic pathway and mLVs using DCE‐MRI after intra‐cisterna magna administration of contrast agent. In comparison to WKY, SHR exhibited reduced clearance of the CSF tracer in six predefined different brain regions, as indicated by significant high levels of PC values (**Figure** **3**A). These findings suggest a delayed drainage of the glymphatic pathway in SHR. To further assess the drainage of mLVs, DCE‐MRI of dCLNs was conducted. **Figure** **4**A‐i showcases representative DCE‐MRI images of the dCLNs in SHR and WKY before and after gadodiamide administration. The representative time‐intensity curves (TICs) of dCLNs derived from DCE‐MRI data revealed that the PC values following 240 min of gadodiamide administration were marked increase (Figure 4A‐ii) and the average time to peak (TTP) value of the dCLNs was significantly extended in SHR compared to WKY (Figure 4A‐iii). In addition, at the 30 min and 60 min marks post‐administration, there was no discernible difference in the PC values between the SHR and WKY groups (*p* > 0.999, both for 30 min and 60 min).

**Figure 5:**
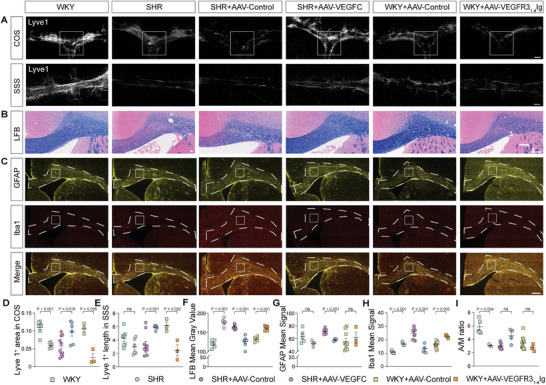
Pathological changes in meningeal lymphatic vessels (mLVs) and corpus callosum (CC) in rats. A) Representative pathological images of mLVs stained with lymphatic vessel endothelial hyaluronan receptor 1 (Lyve1) 647 (in gray) in the meningeal confluence of sinuses (COS) and superior sagittal sinus (SSS), shown in dashed rectangle box. Scale bar = 1000 µm. B) Representative pathological images showing myelin sheath stained with Luxol Fast Blue (LFB) in CC. C) Representative images of glial fibrillary acidic protein (GFAP) (in yellow) and Ionized calcium binding adapter molecule 1 (Iba1) (in red) staining in brain sections. Scale bar = 500 µm. D–E) Quantification of fluorescence intensity, expressed as Lyve1^+^ covered area and length of Lyve1^+^ immunoreactivity in D) COS and E) SSS. Data for SHR: *n* = 6, SHR+AAV‐VEGFC: *n* = 6, SHR+AAV‐Control: *n* = 10, WKY: *n* = 7, WKY+AAV‐VEGFR3_1‐4_‐Ig: *n* = 3, WKY+AAV‐Control: *n* = 3. Pooled data from two independent experiments. F) Quantification of demyelination in CC. Scale bar = 300 µm. SHR showed a significantly decreased optical density (i.e., an increased mean gray value) compared to WKY. Data for SHR: *n* = 5, SHR+AAV‐VEGFC: *n* = 7, SHR+AAV‐Control: *n* = 10, WKY: *n* = 6, WKY+AAV‐VEGFR3_1‐4_‐Ig: *n* = 5, WKY+AAV‐Control: *n* = 5. Pooled data from two independent experiments. G–I) Quantification of fluorescence intensity of G) GFAP, H) Iba1 and GFAP/Iba1 ratio (A/M ratio, I) immunoreactivity, expressed as mean signal in a fixed square region. Data for SHR: *n* = 4, SHR+AAV‐VEGFC: *n* = 4, SHR+AAV‐Control: *n* = 9, WKY: *n* = 5, WKY+AAV‐VEGFR3_1‐4_‐Ig: *n* = 3, WKY+AAV‐Control: *n* = 10. Pooled data from two independent experiments. All error bars represent mean ± s.e.m. *P* values were calculated by two‐tailed unpaired Student's t‐test or Welch's t‐test.

To investigate meningeal lymphangiogenesis in the animal model, whole‐mount meninges from rats were subjected to immunostaining for the lymphatic endothelial cell (LEC) marker, lymphatic vessel endothelial hyaluronan receptor 1 (Lyve‐1). The extent of meningeal lymphangiogenesis was assessed by calculating the Lyve‐1‐positive area in confluence of sinus (COS) and Lyve‐1‐positive length in superior sagittal sinus (SSS). Immunofluorescence analysis exhibited decreased lymphangiogenesis in SHR compared to WKY (**Figure** **5**A,D,E).

To assess astrogliosis and microglia activation, cyro‐sections of the brain were immunolabeled with glial fibrillary acidic protein (GFAP) and Iba1. The distribution of astrocytes and microglia in the corpus callosum (CC) is presented in Figure 5C. Quantification of GFAP and Iba1 distribution was performed by measuring the mean signal in a fixed square region, with the average obtained from three micrographs per animal. We observed a significant increase in the activation of microglia in SHR compared to WKY, while no alterations were found in astrocytes between SHR and WKY (Figure 5G–I). To evaluate the severity of white matter demyelination, Luxol Fast Blue (LFB) staining was used, revealing greater demyelination in the CC of SHR compared to WKY (Figure 5B,F).

### 2.4 Enhancement of the mLVs Drainage Mitigated Neuroinflammation and Ameliorated White Matter Demyelination

**Figure 6:**
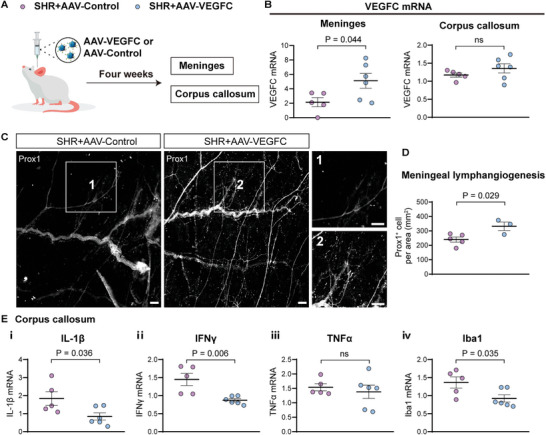
VEGF‐C promotes meningeal lymphangiogenesis and ameliorates neuroinflammation in SHR. A,B) Illustration depicting the administration of A) AAV2/9 and B) subsequent VEGFC mRNA expression in the meninges and corpus callosum (CC). AAV2/9‐VEGF‐C or AAV2/9‐Control was injected into the cisterna magna at a dose of 10^13^ genome copies. Four weeks after injection, samples were collected for mRNA examination. SHR+AAV‐VEGFC: *n* = 6, SHR+AAV‐Control: *n* = 5. C,D) Meningeal lymphangiogenesis after VEGF‐C delivery via AAV in SHR. C) White dots represent Prox1 (a marker of lymphatic endothelial cells). D) The count of Prox1^+^ cells within the region delineated by the middle meningeal artery, superior sagittal sinus, and confluence of sinus served as a measure of meningeal lymphangiogenesis. Scale bars indicate 250 µm for both low magnification and the enhance magnification of areas 1 and 2. SHR+AAV‐VEGFC: *n* = 3, SHR+AAV‐Control: *n* = 5. E) mRNA levels of proinflammatory genes in the CC are detailed. SHR+AAV‐VEGFC: *n* = 6, SHR+AAV‐Control: *n* = 5. All error bars represent mean ± s.e.m. *P* values were calculated by two‐tailed unpaired Student's t‐test or Welch's t‐test.

To gain deeper insights into the implications of impaired drainage of mLVs in white matter demyelination, we proceeded to investigate the effectiveness of enhancing CNS lymphatic drainage in attenuating white matter demyelination. AAV delivery of VEGF‐C has previously been shown to successfully enhance lymphatic function.^[^ ^10^, ^23^, ^24^ ^]^ In this study, we injected AAV2/9‐CMV‐rVEGF‐C or AAV2/9‐CMV‐mNeonGreen (as control) into the cisterna magna of SHR (**Figure** **6**A). Four weeks post i.c.m. viral injection, there was a marked increase in VEGF‐C mRNA expression in the meninges of SHR treated with AAV2/9‐CMV‐rVEGF‐C compared to those treated with the control vector (Figure 6B). In line with the elevated VEGF‐C mRNA expression, a significant enhancement in meningeal lymph angiogenesis was observed in AAV2/9‐CMV‐rVEGF‐C‐treated SHR when compared to the control group (Figures 5A,D,E and 6C,D). Notably, the clearance of the tracer in six predetermined brain regions was significantly faster in the AAV2/9‐CMV‐rVEGF‐C‐treated group compared to the control group (Figure 3B). Moreover, there was no significant difference in glymphatic clearance function between the AAV2/9‐control treated SHR and SHR groups (Figure S1, Supporting Information). DCE‐MRI of dCLNs was also performed in SHR treated with AAV2/9‐CMV‐rVEGF‐C and controls (Figure 4B‐i). Analysis indicated a significant decrease in PC values following 240 min of gadodiamide administration (Figure 4B‐ii), and a significant reduction in the average TTP values of dCLNs in AAV2/9‐CMV‐rVEGF‐C‐treated SHR, compared to the control (Figure 4B‐iii). In addition, at the 30‐minute and 60‐minute marks post‐administration, there was no discernible difference in the PC values between the AAV2/9‐CMV‐rVEGF‐C‐treated SHR and the control (*p* > 0.999, both for 30 min and 60 min). The activation of microglia was significantly reduced in AAV2/9‐CMV‐rVEGF‐C‐treated group compared to the control group, as indicated by decreased Iba1 mean signal and increased GFAP/Iba1 ratio (A/M ratio) (Figure 5C,H,I). In general, VEGF‐C treatment resulted in a reduction of proinflammatory gene expression in the corpus callosum in SHR (Figure 6E). The expression levels of IL‐1β, IFNγ and Iba1 were significantly lower in the corpus callosum of the AAV2/9‐CMV‐rVEGF‐C‐treated group in comparison to the control group, respectively (Figure 6E‐i,ii,iv). TNFα expression levels remained unchanged between the two groups within the corpus callosum (Figure 6E‐iii). LFB staining also revealed that the AAV2/9‐CMV‐rVEGF‐C‐treated group exhibited less demyelination in CC compared to the control group (Figure 5B,F). Taken together, these findings strongly support the notion that overexpression of VEGF‐C enhances meningeal lymphangiogenesis, prompts meningeal lymphatic drainage, ameliorates neuroinflammation and white matter damage in SHR.

### 2.5 Decreased Drainage of mLVs Worsened Neuroinflammation and Aggravated White Matter Demyelination

To investigate the role of mLVs drainage in white matter demyelination, we employed a VEGF‐C/D trap strategy to inhibit the growth of mLVs. Previous studies have demonstrated that a fusion protein consisting of the first four Ig‐homology domains of vascular endothelial growth factor receptor 3 (VEGFR3) and the IgG Fc domain, known as VEGFR3_1‐4_‐Ig, effectively binds to VEGF‐C and VEGF‐D, inhibiting VEGF‐C‐induced VEGFR3 phosphorylation and leading to the regression of mLVs in adult mice.^[^ ^23^, ^25^ ^]^ In this experiment, AAV2/9‐CMV‐rVEGFR3_1‐4_‐Ig or AAV2/9‐CMV‐Flag (as control) was i.c.m. injected into WKY and SHR. In WKY, although some rudimentary mLVs were still occasionally detected around the dural sinuses even 4–6 weeks after AAV2/9‐CMV‐rVEGFR3_1‐4_‐Ig virus injection, there was a significant regression compared to the control group (Figure 5A,D,E). Consistent with these morphological changes, the clearance of the CSF tracer in six predefined brain regions was significantly reduced (Figure 3C). Moreover, there was no significant difference in glymphatic clearance function between the AAV2/9‐control treated WKY and WKY groups (Figure S1, Supporting Information). Similarly, DCE‐MRI of dCLNs was performed in WKY treated with AAV2/9‐CMV‐rVEGFR3_1‐4_‐Ig and controls (Figure 4C‐i). Our findings demonstrated a significant increase in PC values after 240 min of gadodiamide administration (Figure 4C‐ii), and the average TTP values of dCLNs were significantly elevated in AAV2/9‐CMV‐rVEGFR3_1‐4_‐Ig treated WKY compared to the control (Figure 4C‐iii). In addition, at the 30 min and 60 min marks postadministration, there was no discernible difference in the PC values between the AAV2/9‐CMV‐rVEGFR3_1‐4_‐Ig treated WKY and the control (*p* > 0.999, *p* = 0.827). The activation of microglia and the demyelination in CC were significantly increased in the AAV2/9‐CMV‐rVEGFR3_1‐4_‐Ig group compared to the control group (Figure 5B,F,C,H,I). However, we did not observe the aforementioned changes in the AAV2/9‐CMV‐rVEGFR3_1‐4_‐Ig treated SHR compared to the control group (Figure S2, Supporting Information), probably due to their inherent poor lymphangiogenesis state. Taken together, these results strongly indicate that trapping VEGF‐C‐VEGFR3 signaling in the brain promotes brain inflammation and exacerbates white matter damage by inducing regression of mLVS.

### 2.6 Identification of Genes and Pathways in Corpus Callosum Restored by VEGF‐C‐Induced Meningeal Lymphangiogenesis

**Figure 7:**
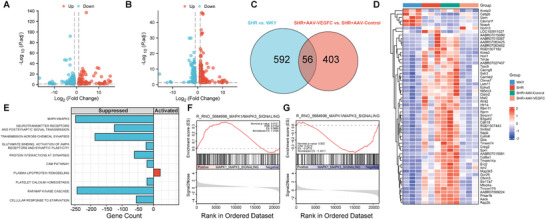
The effect of vascular endothelial growth factor C (VEGF‐C) overexpression on the gene expression profile and functional changes in the corpus callosum. RNA‐seq analysis. *n* = 3 per group. A) Volcano plot displaying a total of 648 significant differentially expressed genes (DEGs) with 204 downregulated genes (blue dots) and 444 upregulated genes (red dots) in the spontaneously hypertensive rats (SHR) versus Wistar‐Kyoto rats (WKY) group. Significant DEGs were considered at a threshold with an absolute value of log_2_ fold change (log2 FC) greater than 1 and an adjust *p*‐value less than 0.05. B) Volcano plot illustrating a total of 459 DEGs with 227 downregulated genes (blue dots) and 232 upregulated genes (red dots) in the AAV2/9‐CMV‐rVEGF‐C treated SHR (SHR+AAV‐VEGFC) versus AAV2/9‐control treated SHR (SHR+AAV‐Control) group. C) Venn diagram revealing intersected genes. D) Heatmap showing DEGs among the groups to identify genes that were altered in SHR but recovered to the levels of the WKY group by VEGF‐C overexpression. E) Gene set enrichment analysis (GSEA) conducted to examine functional changes caused by VEGF‐C overexpression. F) GSEA plot showing the MAPK1/MAPK3 signaling pathway was activated in SHR compared to WKY. NES = 1.319, NOM *p*‐val = 0.012, FDR *q*‐val = 0.215. G) GSEA plot demonstrating the MAPK1/MAPK3 signaling pathway was suppressed after AAV2/9‐CMV‐rVEGF‐C treatment in SHR. NES = ‐1.434, NOM *p*‐val = 0.001, FDR *q*‐val = 0.21.

RNA‐seq analysis identified a total of 648 significant differentially expressed genes (DEGs) with 204 downregulated and 444 upregulated genes (|log_2_FoldChange (FC)| > 1, Adjust *p* < 0.05) in the comparison between SHR and WKY group (**Figure** **7**A). Similarly, the volcano plot demonstrated a total of 459 DEGs with 227 downregulated and 232 upregulated in AAV2/9‐CMV‐rVEGF‐C treated SHR versus AAV2/9‐CMV‐mNeonGreen (as control) treated SHR group (Figure 7B). A Venn diagram showed an overlap of 56 genes (Figure 7C) and their gene expression profiles are presented in Figure 7D. Gene set enrichment analysis (GSEA) revealed that VEGF‐C treatment could activate the plasma lipoprotein remodeling pathway and suppress MAPK1/MAPK3, RAF/MAP kinase cascade, glutamate binding, neurotransmitter receptors and postsynaptic signal transmission, activation of AMPA receptors and synaptic plasticity, transmission across chemical synapses, protein interactions at synapses, platelet calcium homeostasis, and cell adhesion molecule pathways (Figure 7E–G). It is noteworthy that all the above suppressed pathways were activated in the SHR group compared to the WKY. Decreased MAPK1/MAPK3 pathway may also be related to decreased microglia activation and subsequent neuroinflammation by VEGF‐C treatment. Figure S4 (Supporting Information) represents the Gene ontology (GO) and Kyoto Encyclopedia of Genes and Genomes (KEGG) enrichment analysis of significant DEGs in the SHR versus WKY and AAV2/9‐CMV‐rVEGF‐C treated SHR versus control group.

## 3 Discussion

Although increasing evidence speculated about the potential role of mLVs and the glymphatic pathway in WMH,^[^ ^13^, ^26^ ^]^ substantial evidence from human beings is lacking. This novel research utilizing Glymphatic MRI in human directly visualized the delayed clearance of CSF tracers in patients with a high burden of WMH, and further revealed a clear association between the volume of WMH and the drainage function of the mLVs and glymphatic pathway. Furthermore, the causal relationship was confirmed in a longitudinal cohort of individuals with CSVD, demonstrating the predictive ability of glymphatic function in the growth of WMH based on the widely used noninvasive DTI‐ALPS index.

The clinical findings discussed above were substantiated by animal studies. Both SHR and AAV2/9‐CMV‐rVEGFR3_1‐4_‐Ig‐treated (blocked the grow of mLVs) WKY rats exhibited significantly impaired lymphangiogenesis and glymphatic dysfunction when compared to WKY rats. This impairment was accompanied by corresponding demyelination in white matter. In SHR, compromised glymphatic pathway function was observed at both early and advanced stages of hypertension, as revealed by DCE‐MRI.^[^ ^14^ ^]^ Additionally, SHR displayed visibly enlarged perivascular spaces, which is another characteristic feature of CSVD.^[^ ^27^ ^]^ Prior hypotheses proposed that arterial wall stiffening resulting from hypertension could impact vessel wall pulsatility, thereby reducing the efficiency of peri‐arterial pumping, which is a key driver of CSF transport.^[^ ^17^, ^28^ ^]^ Differently, our study unveiled a novel mechanism underlying glymphatic dysfunction in SHR, namely, impairment of mLVs, which represents a downstream component of glymphatic pathway. The impaired mLVs can disrupt CSF glymphatic outflow, as observed in various animal models.^[^ ^24^, ^29^ ^]^ Recently, an intriguing study demonstrated that increased blood pressure in SHR can induce lymphatic endothelial dysfunction in thoracic ducts through elevated oxidative stress mediated by the p38 MAPK/NADPH oxidase pathways.^[^ ^30^ ^]^ In our study, we also observed increased oxidative stress in both SHR and AAV2/9‐CMV‐rVEGFR3_1‐4_‐Ig treated WKY, along with alterations in several inflammation signal pathways in CC, as well as microglia activation. These findings suggest that similar changes may occur in meningeal LECs in response to hypertension. Taken together, these results indicate that impaired drainage of mLVs may serve as a trigger for glymphatic dysfunction and white matter lesions in SHR.

Several previous studies have provided evidence that VEGF‐C could be therapeutic target in brain disease and inflammation disease. Developing a nano‐plumber that coencapsulates the microenvironment regulator pro‐DHA and the lymphatic‐specific growth factor VEGF‐C, Chen et al significantly improves the neurological function of rodents with traumatic brain injury through enhanced glymphatic‐lymphatic drainage, suppressed microglia and astrocytes activation.^[^ ^31^ ^]^ Overexpression of VEGF‐C improves clearance of senescent astrocytes and mitigates neuroinflammation.^[^ ^32^ ^]^ Notably, VEGF‐C also shines a light on brain tumors. Using a mouse model of glioblastoma, Song et al. report that VEGF‐C can improve the efficacy of immune checkpoint blockade by enhancing the priming of anti‐tumor CD8^+^ T cell responses in the draining dCLNs, thus promoting immune surveillance and eradication of tumors.^[^ ^33^ ^]^ Similarly, VEGF‐C overexpression improved the efficacy of radiotherapy in the treatment of brain tumors.^[^ ^34^ ^]^ Together, previous studies have laid the foundation for a new therapeutic strategy to alleviate neuroinflammation and enhance immunosurveillance by VEGF‐C stimulation.

Our study represents the first investigation to underscore the significance of enhanced mLVs and glymphatic pathway in preventing WMH growth, as treatment with AAV2/9‐CMV‐rVEGF‐C was shown to increase meningeal lymphangiogenesis and enhance glymphatic pathway drainage, thereby resulting in reduced brain neuroinflammation and white matter demyelination in animal model of WMH. Neuroinflammation is increasingly recognized as a critical pathology underlying WMH, with supporting evidence stemming from both clinical and preclinical studies.^[^ ^15^, ^35^ ^]^ Notably, our findings provide additional evidence of microglia‐involved neuroinflammation regulated by mLVs in SHR. Indeed, preclinical studies utilizing animal models of WMH have observed enhanced microglial activation, increased oligodendrocyte density, and infiltration of T cells and neutrophils.^[^ ^36^, ^37^ ^]^ Several studies have focused on modulating microglia to alleviate white matter injuries. Yu et al. reported that metformin, rapamycin, and nicotinamide mononucleotide could attenuate white matter lesions by modifying microglial polarization and inhibiting phagocytosis.^[^ ^38^ ^]^ Tan et al. also found that quercetin alleviated demyelination by regulating microglial phenotype transformation in mice with vascular dementia.^[^ ^39^ ^]^ Guo et al. demonstrated that exposure to an enriched environment could promote white matter recovery after stroke by substantially decreasing the level of pro‐inflammatory cytokines induced by microglia activation.^[^ ^40^ ^]^ The association between meningeal lymphangiogenesis and decreased microglia‐mediated neuroinflammation may expand the therapeutic target for WMH and even other neurodegenerative disorders.

Furthermore, to investigate how increased drainage of mLVs ameliorate microglia‐related neuroinflammation, RNA‐seq analysis was conducted, focusing on the CC, in AAV2/9‐CMV‐rVEGF‐C treatment SHR compared to SHR control group. The CC was selected as the primary ROI due to its prominent demyelination and microglial activation in SHR.^[^ ^22^ ^]^ Signal pathways were identified and among them, a significant activation of MAPK1/MAPK3 pathway was observed in SHR, but then reversed by AAV2/9‐CMV‐rVEGF‐C treatment to a level similar to that of control rats. The MAPK1/MAPK3, also known as extracellular signal regulated kinases (ERKs) 2 and 1, are phosphorylated by the MAP2Ks 1 and 2 in response to various extracellular stimuli, promoting cell survival, metabolism, and transcription, among other functions. MAPK1/MAPK3 kinases play a crucial role in inhibiting autophagy by negatively regulating the transcription factor EB. In the context of microglia activation during neuroinflammation, suppression of autophagy is essential to downregulate phagocytic degradation. Moreover, in ERK2‐deficient mice, subsequent inflammatory glial responses in CC were significantly attenuated, characterized by a reduction in microglia accumulation and reactive astrocytes.^[^ ^41^ ^]^ These findings suggest the involvement of MAPK1/MAPK3 pathway in microglia activation. Based on these results, it can be hypothesized that the impaired mLVs in SHR, along with blocked immune cells clearance, may activate the MAPK1/MAPK3 pathway in the brain, subsequently exacerbating microglia‐related neuroinflammation. Modulating the MAPK1/MAPK3 pathway may be a potential therapeutic approach for mitigating inflammatory processes in WMH.

Our results have several clinical implications. Firstly, our clinical findings, demonstrating that glymphatic dysfunction predicts the growth of WMH, provide a promising quantitative imaging biomarker for early‐stage differentiation of high‐risk WMH. The noninvasive, convenient, and cost‐effective nature of the ALPS index may facilitate its acceptance by patients. Secondly, our animal experiments suggest that meningeal lymphangiogenesis is involved in the pathogenesis of neuroinflammation associated with microglia activation, potentially through the MAPK1/MAPK3 pathway. These findings support the role of mLVs and the glymphatic pathway in maintaining immune balance in the CNS. Thirdly, despite the prevalence and detrimental effects of WMH, prevention strategies primarily focused on passive management of known vascular risk factors, yielding limited efficacy for active intervention. With increasing research efforts dedicated to enhancing the drainage of mLVs and glymphatic pathway,^[^ ^10^, ^24^, ^42^, ^43^ ^]^ as well as the development of medicines such as edaravone dexborneol and ulinastatin that alleviate neuroinflammation via the MAPK1/MAPK3 pathway in cerebral injury,^[^ ^44^, ^45^ ^]^ our findings may open up a reliable new avenue for active prevention strategies.

However, there are some limitations to consider. First, due to the availability of contrast agent at the time of data collection, we used gadodiamide for the intrathecal injection in our cohort. Although its contrast enhancement is slightly inferior to gadobutrol, we were still able to detect differences in signal intensity. However, future studies should take into consideration the potential impact of different contrast agents on quantitative measurements. Second, the positioning of the rats may have varied each time when they underwent MRI scanning, although we ensured that the ROIs were consistently drawn in identical locations based on anatomical landmarks. Thirdly, our analysis employed TICs and TTP values to assess the drainage function from mLVs to dCLNs, a method that aligns with approaches utilized in previous studies.^[^ ^46^ ^]^ However, some research has adopted the technique of injecting tracer dye into the cisterna magna to directly evaluate dCLNs function, where impaired mLV drainage is indicated by slow tracer accumulation in the dCLNs.^[^ ^24^, ^47^ ^]^ Given that dCLNs may exhibit variable drainage capacities across different diseases and individuals, a dynamic and multifaceted metric assessment becomes imperative. Future studies aiming for a direct assessment of mLV drainage should endeavor to provide more comprehensive evidence. Fourthly, it is worth noting that the anesthetic drugs utilized in our study could have influenced the glymphatic system, even though the same drugs were administered across various treatment groups. Lastly, we did not measure biomarkers representing inflammation in human, which is a limitation of our study. Future studies are needed to investigate these biomarkers and their potential implications.

In conclusion, our study provides compelling evidence that impaired drainage of mLVs and the glymphatic pathway is specifically linked to the pathogenesis of WMH. The augmentation of meningeal lymphatic function emerges as a promising therapeutic target for delaying or potentially even preventing the growth of WMH. These findings shed light on the underlying mechanisms of WMH development and offer new avenues for therapeutic interventions in WMH‐related conditions.

## 4 Experimental Section

### Ethics Statement

The study, which involved the administration of intrathecal gadolinium agents and CIRCLE Cohort, received approval from the Ethics Committee of The Second Affiliated Hospital of Zhejiang University School of Medicine (Approval no. Yan‐2018‐111 and Yan‐2017‐019). All clinical investigation was conducted in compliance with the principles expressed in the Declaration of Helsinki. All patients were included after informed consent. All animal procedures were approved by the Animal Ethics Committee of The Second Affiliated Hospital of Zhejiang University School of Medicine (Approval no. AIRB‐2021‐1246).

### Participants Underwent Glymphatic Magnetic Resonance Imaging (MRI)

To directly visualize the glymphatic pathway, patients with indications for lumbar puncture due to the need for diagnose during the clinical practice, were recruited. The selection process adhered to strict exclusion criteria, excluding individuals with known adverse reaction to contrast agents, a history of severe allergic reactions in general, renal dysfunction, and pregnant or breastfeeding females. For the purpose of this study, patients without diseases known to affect white matter hyperintensity (WMH), such as neurodegenerative diseases and encephalitis, were included. All recruited patients underwent MRI before lumbar puncture (at baseline) and at multiple time points including 4.5, 15, and 39 h after intrathecal injection of 1 mL gadodiamide (0.5 mmol mL^−1^) during a study period from April 2018 to June 2022. It is worth noting that patients were ensured to maintain their normal sleep patterns throughout the duration of the study, thus preserving a natural sleep‐wake cycle.

### Participants in CIRCLE Cohort

To noninvasively assess the glymphatic function in patients with WMH, a retrospective review of participants enrolled in the CIRCLE study (ClinicalTrials.gov ID: NCT03542734) from January 2010 to June 2022 was conducted. The CIRCLE study is a single‐center prospective observational study that aims to include adults with and without cerebral small vessel disease (CSVD), who are free of known dementia or stroke. Participants in the study undergo neuropsychological testing and multimodal MR scans. In detail, the inclusion criteria for the CIRCLE study were as follows: 1) age greater than 40; 2) absence of known dementia or stroke (at least six months after the onset in patients with acute lacunar stroke). The exclusion criteria encompassed: 1) any contraindications for MRI; 2) a history of severe head injury resulting in loss of consciousness or prior intracranial surgery; 3) presence of cancer.

### Imaging Protocol in Humans

A 3.0T MRI scanner (GE 750; GE Healthcare, Chicago, IL) was used for imaging in this study. The imaging protocol settings remained consistent across all time points. The following sequences were acquired: sagittal head 3D T1‐weighted (3D‐T1), high‐resolution head 2D T2‐fluid attenuated inversion recovery (2D T2‐FLAIR) imaging, diffusion tensor imaging (DTI), susceptibility weighted imaging (SWI), and neck T1 fat‐suppression imaging. It is important to note that efforts were made to maintain consistent positioning for each patient's scans at 4.5, 15, and 39 h, closely resembling the baseline scan. The specific imaging parameters were as follows: 3D‐T1: repetition time = 7.3 ms, echo time = 3.0 ms, flip angle = 8°, thickness = 1 mm, field of view = 25 × 25 cm^2^, matrix = 250 × 250 pixels. T2‐FLAIR: repetition time = 8,400 ms, echo time = 152 ms, flip angle = 90°, thickness = 3 mm, no slice gap, field of view = 18 × 18 cm^2^, matrix = 320 × 320 pixels. DTI: a single shot, diffusion‐weighted spin echo echo‐planar imaging sequence with a maximum *b*‐value = 1000 s mm^−2^, 30 noncollinear directions, and 1 volume acquired without diffusion weighting (*b*‐value = 0 s mm^−2^). Additional parameters include: repetition time = 4600 ms, echo time = 69.3 ms, slice thickness = 2 mm, slice gap = 1 mm, matrix = 160 × 160 pixels, field of view = 26 × 26 cm^2^. SWI: a three‐dimension multi‐echo gradient‐echo sequence with 8 equally spaced echoes. Parameters include: echo time = 4.5 ms [first echo], inter‐echo spacing = 4.5 ms, repetition time = 58 ms, field of view = 24 × 24 cm^2^, matrix size = 256 × 256 pixels, flip angle = 20°, slice thickness = 2 mm with no slice gap, in‐plane spatial resolution = 0.9375 × 0.9375 mm/pixel. Flow compensation was applied during the acquisition. Neck T1 fat‐suppression imaging: repetition time = 535 ms, echo time = 9.1 ms, thickness = 4 mm, slice gap = 0.5 mm, field of view = 22 × 22 cm^2^, matrix = 130 × 100 pixels.

### Intrathecal Administration of Gadodiamide

The contrast agent was intrathecally injected into either the L3‐4 or L4‐5 lumbar intervertebral space. Prior to the injection, the correct position of the syringe tip in the subarachnoid space was confirmed by observing the backflow of cerebrospinal fluid (CSF) from the puncture needle. The contrast agent used was 1 mL of gadodiamide (Omniscan; GE Healthcare) with a concentration of 0.5 mmol mL^−1^. After the needle was removed, patients were instructed to rotate their bodies around the long axis twice and then maintain a supine position for the following 4 h following the intrathecal injection.

### Evaluation of Imaging in Humans

The regions of interest (ROIs), including grey matter, white matter and CSF, were segmented and manually checked using Statistical Parametric Mapping version 12 software package (SPM12; The Wellcome Centre for Human Neuroimaging, London, UK; https://www.fil.ion.ucl.ac.uk/spm/software/spm12/). The sagittal sinus on head 3D‐T1 images as references was selected, as this region typically does not exhibit significant tracer accumulation after the intrathecal injection of gadodiamide.^[^ ^18^, ^19^ ^]^ For each time point, the mean signal unit was recorded for each ROI and calculated the signal unit ratio between ROIs and sagittal sinus. The glymphatic clearance function of each region was defined as the percentage changes (PC) in the signal unit ratio from baseline to 39 h, with lower PC indicating better glymphatic clearance function.

ROIs were delineated on parasagittal dura (PSD) using RadiAnt (Medixant, Poznan, Poland), a viewer for Digital Imaging and Communications in Medicine. The following steps were undertaken: First, PSD was identified as tissue exhibiting intermediate or high signal intensity. On the baseline coronal T2‐FLAIR images, ROIs were placed within the outer perimeter of PSD. Second, PSD was identified at the same position as the baseline on the 4.5, 15, and 39 h images, as the image positions remained consistent during scanning. Subsequently, for each time point, the signal units for PSD were normalized against the vitreous body of the ocular bulb and recorded as the signal unit ratio. The vitreous body of the ocular bulb served as a reference since there was no significant tracer accumulation in the ocular bulb after the intrathecal injection of gadodiamide.^[^ ^48^ ^]^ The clearance function of the PSD was then defined as the PC in the signal unit ratio from baseline to 39 h, with lower PC indicating better clearance function. ROIs were delineated on deep cervical lymph nodes (dCLNs) using RadiAnt. The clearance function of the dCLNs was defined as the PC in the signal unit ratio from baseline to 39 h, with lower PC indicating better clearance function.

WMH was defined as regions in the white matter exhibiting high signal intensity on T2‐weighted imaging and T2‐FLAIR images, while displaying equal or low signal on 3D‐T1 images (distinct from CSF signal). The segmentation of WMH tissue masks was performed automatically in the native space on T2‐FLAIR images using the lesion segmentation tool toolbox within the Statistical Parametric Mapping version 12 software package (SPM12; The Wellcome Centre for Human Neuroimaging, London, UK; https://www.fil.ion.ucl.ac.uk/spm/software/spm12/). To assess the severity of periventricular and deep WMH, the Fazekas scale, ranging from 0 to 3, was employed. The Fazekas scale provides a rating system to evaluate the extent of WMH involvement in these regions.

To noninvasively evaluate the function of the glymphatic system, the diffusion tensor image analysis was utilized along the perivascular space (DTI‐ALPS) index in participants of the CIRCLE cohort. The methodology for calculating the DTI‐ALPS index has been extensively described in previous studies.^[^ ^13^, ^49^, ^50^ ^]^ This index provides a measure of the clearance function of the glymphatic system by evaluating diffusivity within the perivascular space direction. Specifically, the SWI raw data were processed to phase and magnitude images on workstation (ADW4.4, GE). Diffusivity maps in the direction of the *x*‐axis (right‐left), *y*‐axis (anterior‐posterior), *z*‐axis (inferior‐superior), and color‐coded fractional anisotropy (FA) maps were processed on DTI Studio (https://www.mristudio.org). Within the regions where the direction of the deep medullary veins was perpendicular to the ventricle body, two 5‐mm‐diameter ROIs were placed in the areas of the projection fibers and the association fibers on the color‐coded FA map. The diffusivity values were recorded in the directions of the x‐axis (Dx), y‐axis (Dy), and z‐axis (Dz) of ROIs in the projection fibers and association fibers as Dxproj, Dyproj, Dzproj, Dxassoc, Dyassoc, Dzassoc, respectively. The DTI‐ALPS index was then calculated as [(Dxproj + Dxassoc) / (Dyproj + Dzassoc)].

### Animals

Female spontaneously hypertensive rats (SHR) (200–300 g) and female control Wistar‐Kyoto rats (WKY) (200–300 g) aged 26 weeks were obtained from Charles River (Beijing, China). Rats were housed in a controlled environment at a temperature of 22 °C with a 12 h light/dark cycle. They were provided food and water ad libitum. All experimental procedures were carried out in accordance with the recommendations in the Guide for the Care and Use of Laboratory Animals published by the National Institutes of Health (NIH Publications No. 80‐23, revised 1996) and conformed to the Animal Management Rules of China (Documentation 55, 2002, Ministry of Health, China).

### Blood Pressure (BP) Measurement

BP measurements were obtained using a noninvasive tail‐cuff blood pressure system (CODA Monitor, Kent Scientific Corporation, USA). Rats were gently restrained in a fixator for at least 10 min until calm, and the tail was warmed before measurement. For each individual rat, multiple BP readings ranging from 3 to 13 measurements were taken and recorded. These measurements were then averaged to obtain a representative value for the rat's BP.

### Intra‐Cisterna Magna Injections

To perform the surgical procedure for exposing the cisterna magna and administering injections, rats were anesthetized by intraperitoneal injection of 4% pelltobarbitalum natricum dissolved in sterile saline. The skin in the neck region was shaved and cleaned using 70% ethanol to maintain sterility. Ophthalmic solution was applied to the eyes to prevent drying, and the rat's head was securely fixed in a stereotaxic frame. A small incision of approximately 10 mm was made in the skin, and underlying muscle layers were gently retracted to expose the atlantooccipital membrane of the cisterna magna. To ensure the correct positioning, 0.2 µL CSF was withdrawn using a Hamilton syringe connected to a 33‐gauge needle. Subsequently, 1 µL of gadodiamide (0.5 mmol mL^−1^) was slowly delivered into the CSF‐filled cisterna magna compartment at a rate of 1 µL per minute. After the injection, the syringe was left in place for at least 2 min to prevent any back‐flow of CSF. The incision in the neck skin was then sutured, and the rats were allowed to recover in a supine position on a heating pad until they regained full consciousness. Additionally, a subcutaneous injection of ketoprofen at a dosage of 2 mg kg^−1^ was administered for pain relief. Given the circadian rhythmicity of CSF dynamics, injections for drainage function measures are carried out alternately between different groups. All experiments are conducted at comparable times of the day to guarantee that similar circadian reactions will be present.

### Adeno‐Associated Virus (AAV) Delivery

For experiments involving the evaluation of the effect of viral‐mediated expression of recombinant vascular endothelial growth factor C (rVEGF‐C) (NM_053653.2) on meningeal lymphatic vessels (mLVs), a total of 2 µL sterile phosphate‐buffered saline (PBS) solution containing 10^13^ genome copies per mL of AAV2/9‐CMV‐rVEGF‐C (titre: 9.3E+12V.G. mL^−1^, 1:1, Obio Technology), or control AAV2/9‐CMV‐mneonGreen (AAV2/9‐control) (titer: 5.5E+12V.G. mL^−1^, Obio Technology) was administered. To inhibit lymphangiogenesis, rats received 2 µL of recombinant AAV encoding the ligand binding domains 1–4 of vascular endothelial growth factor receptor 3 (VEGFR3), fused to the rat IgG2B Fc domain (AAV2/9‐CMV‐rVEGFR3_1–4_‐Ig)(titer: 1.5E+13 V.G. mL^−1^, 1:2, Obio Technology)^[^ ^51^ ^]^ or control AAV2/9‐CMV‐Flag (AAV2/9‐control) (titer: 2.5E+13V.G. mL^−1^, 1:4, Obio Technology) as previously described.^[^ ^24^ ^]^ Virus together with 1 µL gadodiamide was injected directly into the cisterna magna CSF at a rate of 1 µL min^−1^, following the procedure described in “Intra‐cisterna magna injections”. All virus vectors were aliquoted and stored at ‐80 °C until used in the experiments.

### MRI of Rats

Animals were anesthetized by intraperitoneal injection of 4% pelltobarbitalum natricum at a dose of 0.15 mL per 100 g of body weight. A 3.0T MRI scanner (GE 750; GE Healthcare, Chicago, IL) equipped with a small aperture animal experiment coil was used for imaging. Dynamic‐contrast enhanced 3D‐T1 BRAVO consisting of 20 sagittal slices of the brain were acquired with the following parameters: repetition time of 8.9 ms, echo time of 3.9 ms, and a slice thickness of 1 mm. The acquired images were analyzed using RadiAnt (Medixant, Poznan, Poland), a Digital Imaging and Communications in Medicine viewer. ROIs were placed at various brain regions including the olfactory bulb, cerebral cortex, thalamus, corpus callosum (CC), the third ventricle, the fourth ventricle, and dCLNs at baseline and 30, 60, 90, 120,150, 180, 210, 240 min after intra‐cisterna magna injection of 1 µL gadodiamide (0.5 mmol mL^−1^). Mean signal unit was measured for each ROI and normalized them against references. According to previous studies, vitreous body of the ocular bulb was chosen as reference, since there was no significant tracer accumulation in this region after intrathecal injection of gadodiamide. The glymphatic clearance function of each region was defined as the PC in signal unit ratio from baseline to each time point, with a lower PC indicating better clearance function. T2‐weighted Fast Spin Echo (T2FSE) consisting of 12 coronal slices of the brain were acquired with the following parameters: a repetition time of 3500 ms, an echo time of 82.3 ms, and a slice thickness of 2 mm. The acquired images were subjected to analysis using Fiji software to calculate the volume of cerebral ventricles.

### Tissue Collection and Processing

Following the administration of a lethal dose of anesthetic by intraperitoneal injection of pelltobarbitalum natricum, the rats were transcardially perfused with ice‐cold PBS, followed by 4% paraformaldehyde (PFA). After stripping the skin and muscle from the bone, the entire head was collected and drop fixed in 4% PFA for 24–48 h at 4 °C. The mandibles and nasal bone were then removed, and an incision was made along the lines of the parietal bone from the cisterna magna to the rostral part on both sides. The parietal bone was removed to expose the brains and fixed meninges, which included the dura mater and arachnoid. These brain and fixed meningeal tissues (dura mater and arachnoid) were carefully dissected from the skullcaps using forceps and kept in PBS at 4 °C until further use. The right half of the fixed brains underwent a series of washes with PBS, followed by cryoprotection in 30% sucrose. The brains were then embedded in Tissue‐Plus O.C.T. compound (Thermo Fisher Scientific) and frozen. Using a cryostat (Leica), 25 µm thick sections were sliced from the frozen brains, and these sections were stored in PBS at 4 °C. The other half of the brain was dehydrated using gradient alcohol, embedded in paraffin, and kept at room temperature. Using a rotary microtome (Leica), 4 µm thick sections were sliced from the paraffin‐embedded brains.

### Immunofluorescence, Imaging, and Quantification

The following steps were applied for rat fixed free‐floating brain sections and meningeal whole mounts for immunofluorescence staining. Meningeal whole mounts or brain cryosections were rinsed in PBS and washed with PBS containing 1% Tween‐20 (Sigma‐Aldrich, CAS:9005‐64‐5) (PBST) for 10 min, followed by incubation in QuickBlock Buffer (Beyotime, Cat. No. P0260) for 1 h at room temperature. This blocking step was followed by incubation with appropriate dilutions of primary antibodies: anti‐Lyve‐1 (R&D, Cat. No.AF7939, 1:200, Polyclonal Sheep IgG), anti‐prox1 (R&D, Cat. No.AF2727, 1:100, Polyclonal Goat IgG), anti‐Iba1 (Abcam, Cat. No. ab178847, 1:400, rabbit monoclonal) and anti‐GFAP (Cell Signaling Technology, Cat. No. #3670, 1:400, mouse monoclonal) in QuickBlock Buffer overnight at 4 °C. Tissue were then washed three times for 10 min at room temperature in 1 mL 1% PBST followed by incubation with the appropriate donkey Alexa Fluor 647 anti‐sheep (Abcam, 1:300, Cat. No. Ab150179), donkey Alexa Fluor 555 anti‐goat (Abcam, 1:300, Cat. No. Ab150130), donkey Alexa Fluor 647 anti‐rabbit (Jackson ImmunoResearch Laboratories, 1:400, Cat. No. 711‐605‐152) or donkey Alexa Fluor 555 anti‐mouse (Abcam, 1:400, Cat. No. ab150110) IgG antibodies for 2 h at room temperature in QuickBlock Buffer. The tissue was then washed three times for 10 min with 1% PBST at room temperature and mounted with Antifade Mounting Medium with DAPI (Beyotime, Cat. No. P0131) and glass coverslips. Preparations were stored at 4 °C for no more than one week until images were acquired using Leica DM6B and a 10× objective with 0.4 NA. The exposure time and brightness/contrast of each image were applied equally across all images, and quantitative analysis of images was performed using Fiji software (NIH).

For the assessment of microglia and astrocyte in the CC, three representative brain sections were imaged for each rat using a wide‐field microscope. The mean area fraction was calculated for each sample by a blinded experimenter using Microsoft Excel. Lymphatic ablation and lymphatic regression were measured by dividing the area of Lyve‐1 labeled by the area of the sinus, and lymphatic coverage on confluence of sinus (COS) and superior sagittal sinus (SSS) was calculated separately. In addition, the number of Prox1^+^ cells within the area surrounded by the middle meningeal artery, COS, and SSS were counted by ImageJ. Meningeal lymphangiogenesis was indicated by a total number of Prox1^+^ cells within the meningeal area analyzed.

### Histological Staining, Imaging, Quantification

Standard Luxol fast blue (LFB) staining using a reagent kit (Baso, China, Cat. No. BA4357B) was performed on the processed paraffin blocks. Histological slides were then scanned using the KFBIO automatic digital slide scanning system (Ningbo, Zhejiang province, China). Demyelination in the CC was examined on LFB‐stained sections at coronal section levels corresponding to +2.0 mm and ‐2.0 mm from bregma. The images were converted into an 8‐bit greyscale format (black/white 0/255). ROIs were placed on upper, middle, and lower layer of the CC. The myelin index (mean gray value) of each ROI was automatically determined using Fiji software.

### Quantitative Real‐Time Polymerase Chain Reaction (qPCR)

Total RNA was isolated from the corpus callosum as well as the whole meninges. While still frozen, these samples were ground to powder with a tissue grinder. Total RNA was extracted from powdered samples by using FastPure Cell/Tissue Total RNA Isolation Kit V2 (Vazyme Biotech Co., Ltd.) according to the manufacturer's instructions. Four hundred nanograms of the total RNA were reverse transcribed by using the HiScript III RT SuperMix for qPCR (+gDNA wiper) (Vazyme Biotech Co., Ltd.). qPCR was performed with Taq Pro Universal SYBR qPCR Master Mix (Vazyme Biotech Co., Ltd.) and analyzed by the QuantStudio 5 Real‐Time PCR Systems (Thermo Fisher Scientific). The oligonucleotide primers were all custom designed and synthesized by Ribobio (Guangzhou, China), whose sequences are shown in Table S11 (Supporting Information). Relative mRNA expression levels were calculated based on the 2^−ΔΔ^CT method and normalized to the β‐actin expression.

### RNA Extraction, Sequencing, and Analysis

Rats were anesthetized by intraperitoneal injection of pelltobarbitalum natricum, followed by transcardially perfusing with ice‐cold PBS. The brains were then extracted from the cranial cavity, and the cerebellum and olfactory bulbs were removed. The brains were sectioned using a rat brain mold, and the CC was rapidly isolated under a stereomicroscope using ophthalmic tweezers. The CC samples were immediately snap‐frozen in dry ice and stored at ‐80 °C until further use. Total RNA was extracted from the samples using TRIzol reagent (Thermo Fisher Scientific, 15596018), and the purity, quality, and integrity of the samples were assessed. Library construction was carried out by LC‐Bio Technology Co., Ltd. (Hangzhou, China). Subsequently, 2 × 150 bp paired‐end sequencing (PE150) was performed on the Illumina Novaseq 6000 platform. After preprocessing, the high‐quality reads were aligned to the rat reference genome by HISAT2 software (https://daehwankimlab.github.io/hisat2/). Differentially expressed genes (DEGs) were identified based on a fold change (FC) >2 or fold change < 0.05 criteria. Gene Ontology (GO) and Kyoto Encyclopedia of Genes and Genomes (KEGG) pathway enrichment analyses were conducted. Gene set enrichment analysis (GSEA) was performed using the GSEA v4.3.0 software (Broad Institute) with 1000 permutations, utilizing annotations from the Reactome pathways. All sequencing services were provided by LC Biotech Corporation (Hangzhou, China). Significantly differentially expressed mRNAs were selected based on criteria of |Log_2_FC| > 1 and adjusted *P* value < 0.05. GO and KEGG pathway enrichment analyses were performed using the OmicStudio tools at https://www.omicstudio.cn/tool. In GSEA, a false‐discovery rate (FDR) < 0.25 was considered significant. Heatmaps of significant DEGs and GO/KEGG enrichment terms were visualized using the R package ggplot2. The raw sequence data were submitted to the NCBI Gene Expression Omnibus datasets with accession number GSE240229.

### Statistical Analysis

In the human study, continuous data were reported as mean with standard deviation (mean ± SD), while categorical data were presented as numbers and percentages. To assess statistical differences among demographic factors, clinical characteristics, and imaging parameters, unpaired t‐test, Mann‐Whitney U test, chi‐square test, or Fisher's exact test, were used as appropriate. To examine correlations between WMH volume and other demographic or imaging parameters, Pearson's correlation or Spearman's correlation analyses were performed. Age and other potential confounders identified in the univariate analyses (*p* < 0.1) or considered of clinical significance (e.g., follow‐up period) were included in the multivariable linear regression models. All statistical analyses were conducted using SPSS software version 25.0 (IBM, Armonk, NY, USA), with significance set at *p* < 0.05 level (two‐tailed). In the animal studies, data are presented as the mean with standard error mean (mean ± s.e.m.). To determine specific differences (e.g., the intensity of ionized calcium binding adapter molecule 1 (Iba1) immunoreactivity) between two groups, a two‐tailed unpaired Student's t‐test was employed, while Welch's t‐test was employed if the assumption of variance homogeneity was violated. For analyzing drainage function during DCE‐MRI procedures among groups, repeated measures two‐way ANOVA with Bonferroni's post hoc tests were utilized. All statistical analyses were performed using Prism 8.0 (GraphPad Software, La Jolla, USA) and statistical significance was indicated as *p < 0.05, **p < 0.01, or ***p < 0.001. No sample outliers were excluded. Statistical tests used for group comparisons in RNA sequencing experiment analysis are specified in the respective methods’ sections.

## Conflict of Interest

The authors declare no conflict of interest.

## Author Contributions

Y.Z., R.X., and Y.L. contributed equally to this work. Y.Z., R.X., Y.L., and M.L. contributed to the conception and design of the study; M.L. coordinated the whole project; Y.Z., K.Z., and M.L. were responsible for the initial assessment and diagnosing of patients; M.F. and C.C. contributed to image acquisition; R.X., Y.L., Y.C., W.R., Z.L., K.Z., R.Z., and J.W. performed image analysis. R.X., Y.L., and J.L. performed immunostaining; R.X., Y.L., W.R., and Z.L. provided statistical analysis and technical support; Y.Z., R.X., Y.L., and M.L. participated in final data analysis and interpretation; Y.Z., R.X., and Y.L. carried out most of the writing with input from other authors. All authors discussed the results and commented on the manuscript.

## Supporting information

Supporting Information

## Data Availability

The data that support the findings of this study are available from the corresponding author upon reasonable request.
