# Supplementary material for: Impaired Meningeal Lymphatics and Glymphatic Pathway in Patients with White Matter Hyperintensity
Source: Adv Sci (Weinh). 2024 May 5;11(26):2402059. doi: 10.1002/advs.202402059 (PMC11234435; doi:10.1002/advs.202402059)
Supplement: Supplementary file 1 — Supporting Information [file ADVS-11-2402059-s001.pdf]

ADVANCED SCIENCE
Open Access

Supporting Information

for *Adv. Sci.*, DOI 10.1002/advs.202402059

Impaired Meningeal Lymphatics and Glymphatic Pathway in Patients with White Matter Hyperintensity

*Ying Zhou, Rui Xue, Yifei Li, Wang Ran, Yuping Chen, Zhongyu Luo, Kemeng Zhang, Ruoxia Zhang, Junjun Wang, Mengmeng Fang, Cong Chen and Min Lou**

Supplemental Material

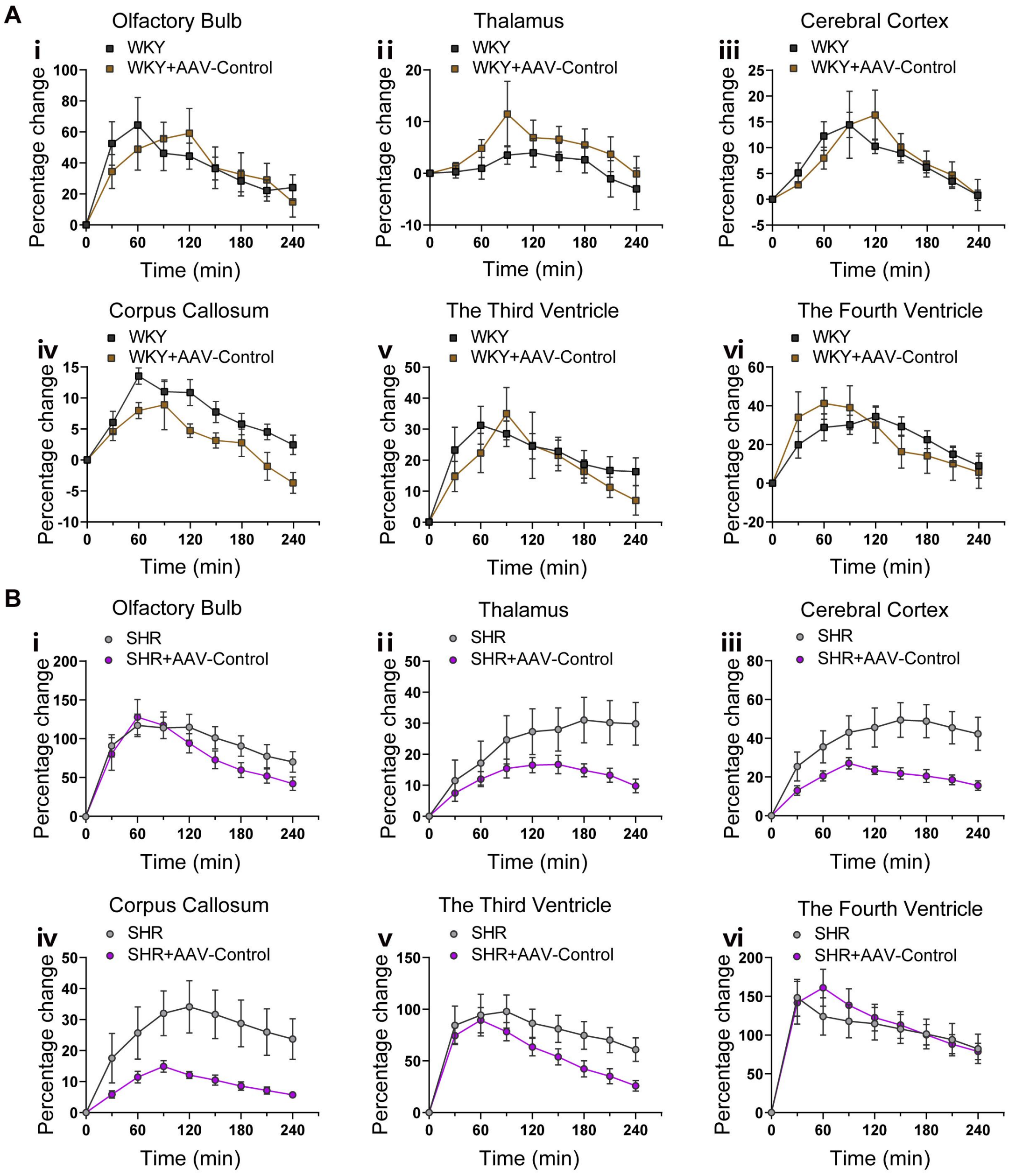

Figure S1. Quantitative assessments of drainage function of glymphatic pathway among AAV2/9-control treated WKY (WKY+AAV-Control) and AAV2/9-control treated SHR (SHR+AAV-Control) compared to WKY and SHR, respectively.

A-B. Dynamic Contrast-Enhanced MRI (DCE-MRI) indicated no significant alterations of percentage changes in olfactory bulb (i), thalamus (ii), cerebral cortex (iii), corpus callosum (iv), the third ventricle (v) and the fourth ventricle (vi) among WKY+AAV-Control and SHR+AAV-Control compared to WKY and SHR, respectively. Data for WKY+AAV-Control: n = 5, SHR+AAV-Control: n = 8, WKY: n = 12, SHR: n = 19. Pooled data from three independent experiments. All error bars

represent mean ± s.e.m. *P* values were calculated by repeated-measures two-way ANOVA with Bonferroni' s post hoc test.

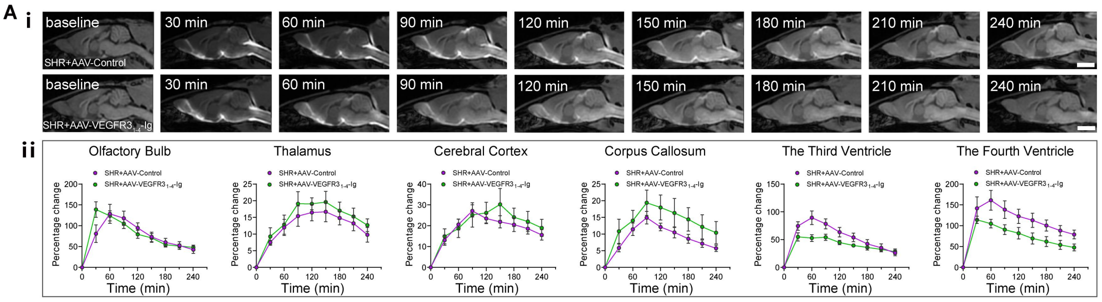

Figure S2. Quantitative assessments of drainage function of glymphatic pathway among AAV2/9-control treated SHR (SHR+AAV-Control) and AAV2/9-CMV-rVEGFR3$_{1-4}$-Ig treated SHR (SHR+AAV-VEGFR3$_{1-4}$-Ig).

Ai. Representative images of the glymphatic pathway in SHR at baseline and every 30 minutes after intrathecal administration of AAV2/9-CMV-Flag (control virus) and AAV2/9-CMV-rVEGFR3$_{1-4}$-Ig. Dynamic Contrast-Enhanced MRI (DCE-MRI) was conducted until 4-6 weeks of viral expression. Scale bar = 5 mm. Aii. No significant alterations of percentage changes in olfactory bulb, thalamus, cerebral cortex, corpus callosum, the third ventricle and the fourth ventricle in SHR+AAV-VEGFR3$_{1-4}$-Ig compared to SHR+AAV-Control. Data for SHR+AAV-VEGFR3$_{1-4}$-Ig: n = 8, SHR+AAV-Control: n = 8. Pooled data from two independent experiments. All error bars represent mean ± s.e.m. *P* values were calculated by repeated-measures two-way ANOVA with Bonferroni' s post hoc test.

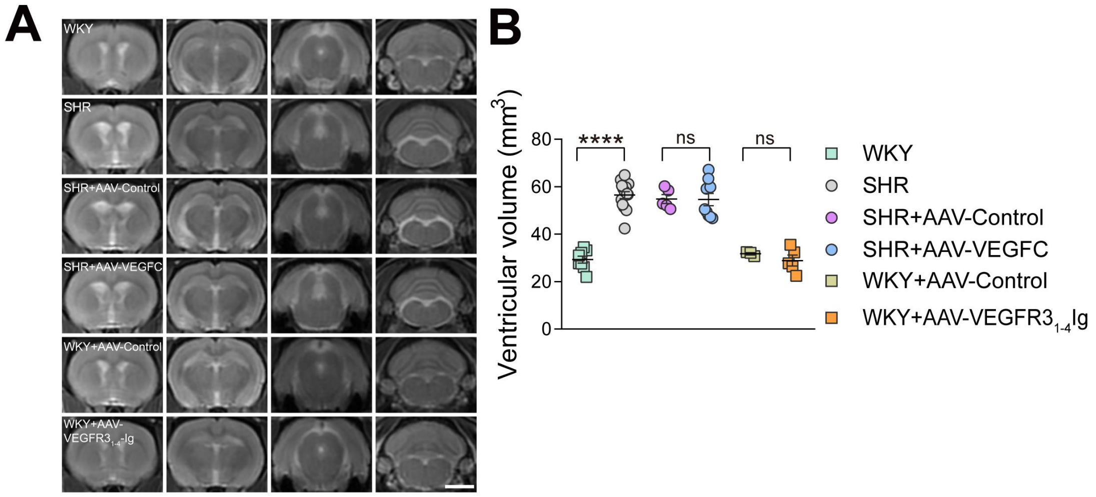

Figure S3. Quantitative assessments of cerebral ventricle volume. A. Representative T2 images of the cerebral ventricle system in WKY, SHR, AAV2/9-control treated SHR (SHR+AAV-Control), AAV2/9-CMV-rVEGF-C treated SHR (SHR+AAV-VEGFC), AAV2/9-control treated WKY (WKY+AAV-Control) and AAV2/9-CMV-rVEGFR3$_{1-4}$-Ig treated WKY (WKY+AAV-VEGFR3$_{1-4}$-Ig). Scale bar = 5 mm. B. Statistical analysis of cerebral ventricle volume between WKY and SHR, SHR+AAV-Control and SHR+AAV-VEGFC, WKY+AAV-Control and WKY+AAV-VEGFR3$_{1-4}$-Ig (two-tailed unpaired Student's t-test). Data for SHR: n = 15, WKY: n = 8, SHR+AAV-VEGFC: n = 9, SHR+AAV-Control: n = 5, WKY+AAV-VEGFR3$_{1-4}$-Ig: n = 5, and WKY+AAV-Control: n = 3. Pooled data from two independent experiments. All error bars represent mean ± s.e.m. ****$p < 0.0001$. ns, not significant.

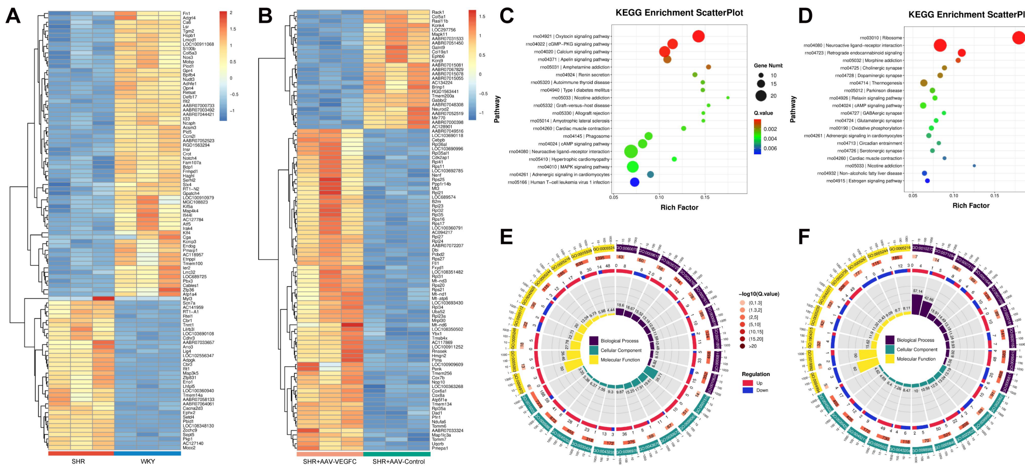

Figure S4. Transcriptomics analysis of SHR versus WKY and AAV2/9-CMV-rVEGF-C treated SHR (SHR+AAV-VEGFC) versus AAV2/9-control treated SHR (SHR+AAV-Control). A-B. Heatmaps showing the expression profiles of the top 100 differentially expressed genes (DEGs) in SHR versus WKY (A) and SHR+AAV-VEGFC versus SHR+AAV-Control (B). C-D. Kyoto Encyclopedia of Genes and Genomes (KEGG) enrichment scatter plot comparing the significantly enriched pathways in SHR versus WKY (C) and SHR+AAV-VEGFC versus SHR+AAV-Control (D). E-F. Gene Ontology (GO) circle plot displaying the enriched GO terms in SHR versus WKY (E) and SHR+AAV-VEGFC versus SHR+AAV-Control (F).

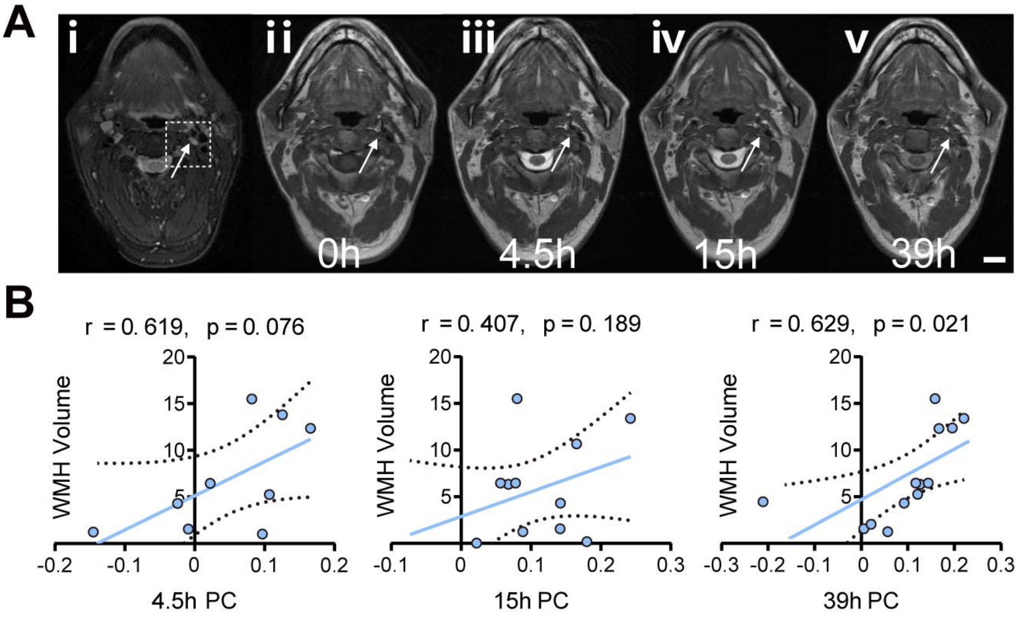

Figure S5. Quantitative assessments of drainage function of meningeal lymphatics (mLVs) to deep cervical lymph nodes (dCLNs) after intrathecal administration of gadodiamide in humans. A. Representative images of the measurement of percentage change (PC) in dCLNs. T2-weighted image (i) was used to determine the location of dCLNs (white dashed box), and the PC of dCLNs (white arrow) was measured on T1-fat-suppression images before (0 h) and 4.5 h, 15 h, and 39 h after intrathecal administration of gadodiamide ((ii)-(iv)). Scale bar = 2 cm. B. Correlation between the WMH volume and the PC value in dCLNs at different time points (4.5h PC group: n = 9, 15h PC group: n = 12, 39h PC group: n = 13, Pearson correlation analysis).

Table S1. Demographics, clinical and imaging data in patients who underwent Glymphatic magnetic resonance imaging

Characteristics	Value
Age (y)	58.30 ± 12.94
Female	27 (50.0)
Smoking	19 (35.2)
Alcohol drinking	13 (24.1)
Hypertension	26 (48.1)
Diabetes mellitus	23 (42.6)
Hyperlipidemia	7 (13.0)
Grey matter volume (ml)	576.09 ± 99.60
White matter volume (ml)	462.11 ± 65.50
Cerebral ventricle volume (ml)	424.60 ± 106.69
WMH volume (ml)	5.60 ± 5.20
Fazekas score	2 (2-4)

WMH: white matter hyperintensity.

Table S2. Linear regression analysis for WMH volume in patients who underwent Glymphatic magnetic resonance imaging

	β	*P* Value		β	*P* Value		β	*P* Value
**4.5h PC PSD**	0.093	0.468	**15h PC PSD**	0.247	0.036	**39h PC PSD**	0.234	0.046
Age	0.682	<0.001	Age	0.446	0.001	Age	0.406	0.002
Female	0.222	0.086	Female	0.280	0.017	Female	0.277	0.015
Hypertension	0.044	0.761	Hypertension	0.211	0.093	Hypertension	0.221	0.077
**4.5h PC Grey matter**	0.273	0.025	**15h PC Grey matter**	0.286	0.014	**39h PC Grey matter**	0.315	0.011
Age	0.417	0.002	Age	0.422	0.001	Age	0.384	0.004
Female	0.151	0.202	Female	0.176	0.124	Female	0.137	0.244
Hypertension	0.156	0.225	Hypertension	0.131	0.297	Hypertension	0.114	0.363
**4.5h PC White matter**	0.173	0.172	**15h PC White matter**	0.225	0.061	**39h PC White matter**	0.266	0.037
Age	0.386	0.007	Age	0.390	0.004	Age	0.380	0.005
Female	0.197	0.105	Female	0.199	0.087	Female	0.155	0.198
Hypertension	0.150	0.259	Hypertension	0.135	0.298	Hypertension	0.111	0.386
**4.5h PC CSF**	0.291	0.013	**15h PC CSF**	0.256	0.028	**39h PC CSF**	0.242	0.044
Age	0.445	0.001	Age	0.438	0.001	Age	0.411	0.002
Female	0.159	0.170	Female	0.190	0.099	Female	0.184	0.115
Hypertension	0.136	0.280	Hypertension	0.114	0.370	Hypertension	0.106	0.408

WMH: white matter hyperintensity; PC: percentage change; PSD: parasagittal dura; CSF: cerebrospinal fluid.

Table S3. Demographics, clinical and imaging data in patients with WMH and controls

Characteristics	WMH (N=1149)	Controls (N=48)	*P* Value
Age (y)	61.92 ± 8.46	54.23 ± 7.01	<0.001
Female	522 (45.7)	14 (29.2)	0.026
Smoking	379 (34.5)	23 (47.9)	0.064
Alcohol drinking	442 (40.4)	24 (50.0)	0.230
Hypertension	734 (65.4)	25 (53.2)	0.118
Diabetes mellitus	226 (20.1)	9 (19.1)	1.000
Hyperlipidemia	345 (30.9)	15 (31.9)	0.873
Grey matter volume (ml)	643.94 ± 71.55	677.56 ± 57.58	0.001
White matter volume (ml)	451.38 ± 56.14	468.73 ± 52.73	0.036
Cerebral ventricle volume (ml)	360.79 ± 101.30	286.86 ± 67.29	<0.001
WMH volume (ml)	7.66 ± 13.10	-	-
Fazekas score	3 (2-5)	-	-
ALPS index	1.49 ± 0.24	1.61 ± 0.26	0.001

WMH: white matter hyperintensity; ALPS: diffusion tensor image analysis along the perivascular space.

Table S4. Multivariate regression analysis for WMH volume, NAWM-FA and NAWM-MD in patients with WMH

	β	P Value		β	P Value		β	P Value
**WMH volume**			**NAWM-FA**			**NAWM-MD**		
ALPS Index	-0.217	<0.001	ALPS Index	0.239	<0.001	ALPS Index	-0.269	<0.001
Age	0.393	<0.001	Age	-0.083	0.006	Age	0.112	<0.001
Hypertension	0.069	0.007	Hypertension	0.004	0.897	Hypertension	-0.052	0.072
Hyperlipidemia	-0.080	0.002	Hyperlipidemia	0.052	0.080	Hyperlipidemia	-0.054	0.063
Alcohol drinking	-0.096	0.001	Alcohol drinking	0.122	<0.001	Alcohol drinking	-0.078	0.013
Cerebral ventricle volume	0.065	0.013	Cerebral ventricle volume	-0.101	0.001	Cerebral ventricle volume	0.108	<0.001
Diabetes mellitus	0.025	0.340	Diabetes mellitus	-0.009	0.767	Diabetes mellitus	-0.043	0.133
Smoking	0.026	0.369	Smoking	-0.029	0.361	Smoking	-0.020	0.535

WMH: white matter hyperintensity; ALPS: diffusion tensor image analysis along the perivascular space; NAWM-FA: normal appearing white matter-fractional anisotropy; NAWM-MD: normal appearing white matter-mean diffusivity.

Table S5. Demographics, clinical and imaging data in patients with and without follow-up T2-FLAIR

Characteristic	WMH patients with follow-up T2-FLAIR (N=191)	WMH patients without follow-up T2-FLAIR (N=958)	*P* Value
Age (y)	63 ± 9	62 ± 8	0.065
Female	92 (48.7)	430 (45.1)	0.380
Smoking	49 (27.4)	330 (35.9)	0.032
Alcohol drinking	49 (27.4)	393 (42.9)	<0.001
Hypertension	116 (63.4)	618 (65.7)	0.553
Diabetes mellitus	33 (18.0)	193 (20.6)	0.481
Hyperlipidemia	37 (20.2)	308 (33.0)	<0.001
WMH volume (ml)	10.98 ± 14.66	6.99 ± 12.67	<0.001
ALPS index	1.41 ± 0.18	1.50 ± 0.24	<0.001
Follow-up period (y)	1.23 ± 0.51	-	-

WMH: white matter hyperintensities; T2-FLAIR: T2-fluid attenuated inversion recovery; ALPS: diffusion tensor image analysis along the perivascular space.

Table S6. Multivariate regression analysis for growth volume of WMH in patients with follow-up T2-FLAIR image

	**β**	***P* Value**
ALPS Index	-0.203	0.007
Age	0.061	0.410
Cerebral ventricle volume	0.037	0.609
Follow-up period	-0.158	0.028

WMH: white matter hyperintensities; ALPS: diffusion tensor image analysis along the perivascular space.

Table S7. Correlation analysis between WMH Volume and age and MRI Measurements in two cohorts

	Patients who underwent Glymphatic MRI		WMH Patients in CIRCLE Cohort	
	**Pearson r or Spearman r**	***P* Value**	**Pearson r or Spearman r**	***P* Value**
Age	0.525	<0.001	0.470	<0.001
Grey matter volume	-0.184	0.183	-0.399	<0.001
White matter volume	0.005	0.973	-0.028	0.351
Cerebral ventricle volume	0.200	0.148	0.125	<0.001
Fazekas score	0.913	<0.001	0.722	<0.001
39h PC PSD	0.380	0.008	-	-
39h PC Grey matter	0.473	<0.001	-	-
39h PC White matter	0.457	<0.001	-	-
39h PC CSF	0.389	0.004	-	-
ALPS Index	-	-	-0.360	<0.001

WMH: white matter hyperintensity; PC: percentage change; PSD: parasagittal dura; CSF: cerebrospinal fluid; ALPS: diffusion tensor image analysis along the perivascular space.

Table S8. Univariate comparison of WMH Volume among factors in two cohorts

	Patients who underwent Glymphatic MRI			WMH Patients in CIRCLE Cohort		
	**Yes**	**No**	**P Value**	**Yes**	**No**	***P* Value**
Female	7.10 ± 5.10	4.10 ± 4.94	0.032	7.66 ± 13.01	7.68 ± 13.24	0.979
Smoking	4.00 ± 4.97	6.58 ± 5.21	0.086	6.32 ± 11.88	7.83 ± 12.98	0.061
Hypertension	7.52 ± 5.34	3.81 ± 4.44	0.007	8.53 ± 13.70	5.55 ± 10.46	<0.001
Diabetes mellitus	6.07 ± 5.54	5.2 5± 4.99	0.573	8.82 ± 15.57	7.16 ± 11.93	0.081
Hyperlipidemia	9.51 ± 6.47	5.02 ± 4.79	0.032	5.87 ± 10.83	8.11 ± 13.39	0.006
Alcohol drinking	5.03 ± 5.93	5.78 ± 5.01	0.657	4.89 ± 5.81	8.93 ± 14.57	<0.001

WMH: white matter hyperintensity.

Table S9. Correlation analysis among growth volume of WMH and MRI measurements in patients with Follow-up T2-FLAIR image

	**Pearson r or Spearman r**	***P* Value**
Age	0.357	<0.001
Grey matter volume	-0.362	<0.001
White matter volume	0.011	0.885
Cerebral ventricle volume	0.165	0.022
Fazekas score	0.793	<0.001
ALPS Index	-0.301	<0.001
Follow-up period	0.073	0.313

WMH: white matter hyperintensity; ALPS: diffusion tensor image analysis along the perivascular space； T2-FLAIR: T2-fluid attenuated inversion recovery.

Table S10. Univariate comparison of growth volume of WMH among factors in patients with follow-up T2-FLAIR image

	Yes	No	P Value
Female	0.46 ± 2.12	1.18 ± 6.29	0.301
Smoking	0.32 ± 3.72	1.00 ± 5.23	0.408
Hypertension	0.68 ± 5.24	1.13 ± 3.99	0.545
Diabetes mellitus	0.82 ± 5.45	0.85 ± 4.68	0.979
Hyperlipidemia	1.15 ± 2.89	0.77 ± 5.20	0.666
Alcohol drinking	0.55 ± 2.69	0.92 ± 5.47	0.653

WMH: white matter hyperintensity; T2-FLAIR: T2-fluid attenuated inversion recovery.

Table S11. Primer sequences

Gene name	GeneBank accession no.	Primer sequences (5'-3')	Expected size (bp)	Annealing temperature for qPCR (°C)
Iba1	NM_017196.3	F: TGACTTTCTCAGAATGATGCTGG<br>R: CAACTCAGAAATAGCTTTCTTGGC	127	60
IL1β	NM_031512.2	F: TGGGATGATGACGACCTGCT<br>R: TGGCTTATGTTCTGTCCATTGAG	143	60
IFNγ	NM_138880.3	F: CAACCCACAGATCCAGCACA<br>R: TCAGCACCGACTCCTTTTCC	103	60
TNFα	NM_012675.3	F: CACCACGCTCTTCTGTCTACTGA<br>R: CGAGTTTTGAGAAGATGATCTGAGT	130	60
VEGFC (rat)	NM_053653.2	F: TGCCAATCACACTTCCTGCC<br>R: GCTGCCTGACACTGTGGTAATGT	108	60
β-actin	NM_031144.3	F: TCAAGATCATTGCTCCTCCTGAG<br>R: ACATCTGCTGGAAGGTGGACA	87	60

Bp: base pair; qPCR: quantitative polymerase chain reaction; F: forward; R: reverse.
